# Impaired climbing and flight behaviour in *Drosophila melanogaster* following carbon dioxide anaesthesia

**DOI:** 10.1038/srep15298

**Published:** 2015-10-19

**Authors:** Nathan R. Bartholomew, Jacob M. Burdett, John M. VandenBrooks, Michael C. Quinlan, Gerald B. Call

**Affiliations:** 1 Arizona College of Osteopathic Medicine (AZCOM), Midwestern University, Glendale, AZ, 85308, USA; 2 Department of Physiology, AZCOM, Midwestern University, Glendale, AZ 85308, USA; 3 Department of Pharmacology, AZCOM, Midwestern University, Glendale, AZ 85308, USA

## Abstract

Laboratories that study *Drosophila melanogaster* or other insects commonly use carbon dioxide (CO_2_) anaesthesia for sorting or other work. Unfortunately, the use of CO_2_ has potential unwanted physiological effects, including altered respiratory and muscle physiology, which impact motor function behaviours. The effects of CO_2_ at different levels and exposure times were examined on the subsequent recovery of motor function as assessed by climbing and flight assays. With as little as a five minute exposure to 100% CO_2_, *D. melanogaster* exhibited climbing deficits up to 24 hours after exposure. Any exposure length over five minutes produced climbing deficits that lasted for days. Flight behaviour was also impaired following CO_2_ exposure. Overall, there was a positive correlation between CO_2_ exposure length and recovery time for both behaviours. Furthermore, exposure to as little as 65% CO_2_ affected the motor capability of *D. melanogaster.* These negative effects are due to both a CO_2_-specific mechanism and an anoxic effect. These results indicate a heretofore unconsidered impact of CO_2_ anaesthesia on subsequent behavioural tests revealing the importance of monitoring and accounting for CO_2_ exposure when performing physiological or behavioural studies in insects.

Immobilisation of flying insects is a necessary laboratory procedure for examining various phenotypes under a light microscope. Several anaesthetic methods can be used to paralyze insects, including the use of common gaseous anaesthetics such as ether, isoflurane, or immobilisation via cold anaesthesia^1^. However, of all the various methods to halt insect activity, carbon dioxide (CO_2_) anaesthesia is the most common among researchers because of its ease of use, effectiveness at immobilisation and affordability^2^. Carbon dioxide anaesthesia is routinely used for insect colony maintenance, surgery, sexing and species identification^3^. After *Drosophila melanogaster* have been anaesthetized with CO_2_, upon removal of the CO_2_ and re-exposure to ambient air, flies return to an upright position and begin seemingly normal activity with no apparent negative effects. However, with new insights on how CO_2_ interacts with specific receptors associated with neurons in *D. melanogaster* and subsequent signalling pathways, questions on whether CO_2_ anaesthesia may be adversely affecting *D. melanogaster* physiology and behaviour are being re-examined^4^.

Carbon dioxide is frequently used to manipulate *D. melanogaster*, although the overall impact of CO_2_ on the behaviour and physiology of these insects is poorly understood^3^,^5^. Numerous reproductive and behavioural traits have been shown to be negatively affected by CO_2_, some immediately following exposure and others lasting several hours or days after the initial exposure^2^,^3^,^6^. Carbon dioxide exposure has been shown to reduce *D. melanogaster* longevity^7^, mating success^2^ and increase resistance to subsequent isoflurane anaesthesia^8^. Woodring *et al.* showed that CO_2_ and anoxia affect aspects of growth, feeding, development, reproduction and behaviour in the cricket, *Acheta domesticus*^9^. Exposure to CO_2_ disturbs normal development and affects movement in the German cockroach, *Blattella germanica*^10^,^11^, blocks glutamate receptors at neuromuscular junctions of *D. melanogaster*^12^ and has widespread effects on the nervous system of the crayfish, *Procambarus clarkii*^13^. Carbon dioxide anaesthesia also increases *D. melanogaster* haemolymph acidity and causes a reduction in heart rate^12^. However, there have only been a limited number of behavioural studies of the effects of CO_2_ anaesthesia in *D. melanogaster*, and much remains to be explored.

Use of 100% CO_2_ to anaesthetize insects not only exposes them to high levels of CO_2_, but simultaneously exposes these organisms to a completely anoxic environment. Thus, exposure to pure CO_2_ impairs or eliminates oxygen (O_2_) delivery to the tissues, likely compromising important energy production pathways. Insects in general are very tolerant to hypoxia and/or anoxia for prolonged periods of time, but their responses vary significantly^14^,^15^. *Drosophila melanogaster*, for example, can tolerate four hours of pure nitrogen (N_2_) exposure and survive^16^. In contrast, *A. domesticus* may experience significant physiological disruption after only a few minutes of anoxia^9^. Prolonged anoxic conditions conjoined with the overall harsh chemical nature of CO_2_ may result in long lasting or permanent changes that may have not been previously accounted for. Due to the large amount of behavioural and physiological studies performed using insects as model organisms, it is of extreme importance to study the effects of CO_2_ and anoxia on insects as this may confound data obtained in these studies. For appropriate selection of an anaesthetic method to use in insect behavioural and physiological studies, it is important to determine if anoxia and CO_2_ anaesthesia have separate effects and how persistent these effects are. This study determined the effects of CO_2_, hypoxia and anoxia on *D. melanogaster* climbing and flight behaviours, which are routinely used to assay motor function and performance in flies.

## Results

### *Drosophila melanogaster* climbing and flight is inhibited by CO_2_ exposure

**Figure 1:**
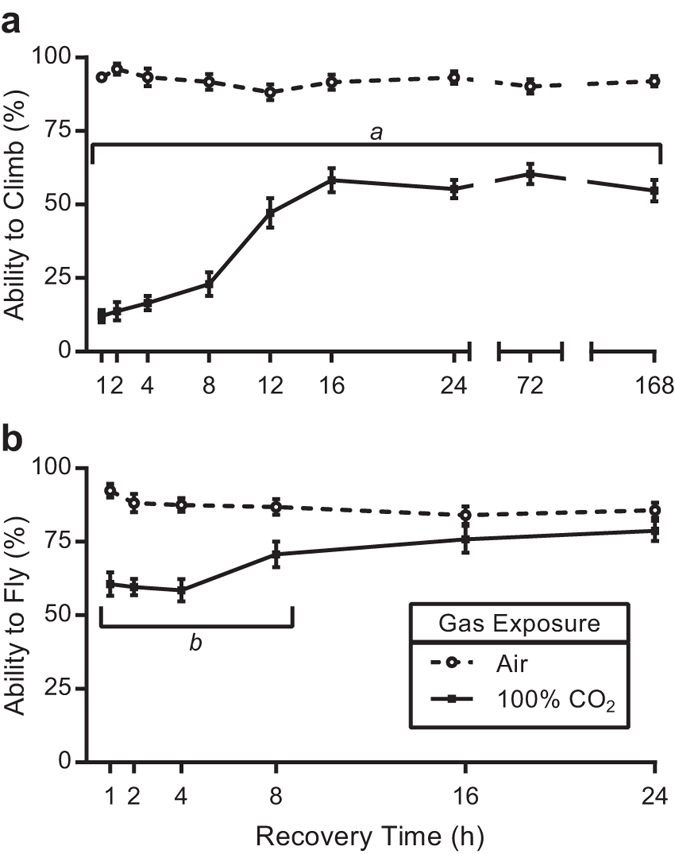
*Drosophila melanogaster* climbing and flight abilities are reduced following a 10 minute exposure to 100% CO_2_. Flies were exposed to 100% CO_2_ for 10 minutes and allowed to recover for varying times and assayed for the ability to (**a**) climb and (**b**) fly. CO_2_ inhibited climbing at all recovery time points assayed while flight was only reduced through 8 hours. Data points represent the mean ± SEM of the percentage of flies able to climb or fly. Statistical analysis was performed by Student’s *t*-tests. *a* = P < 0.001, *b* = P < 0.01.

**Figure 2:**
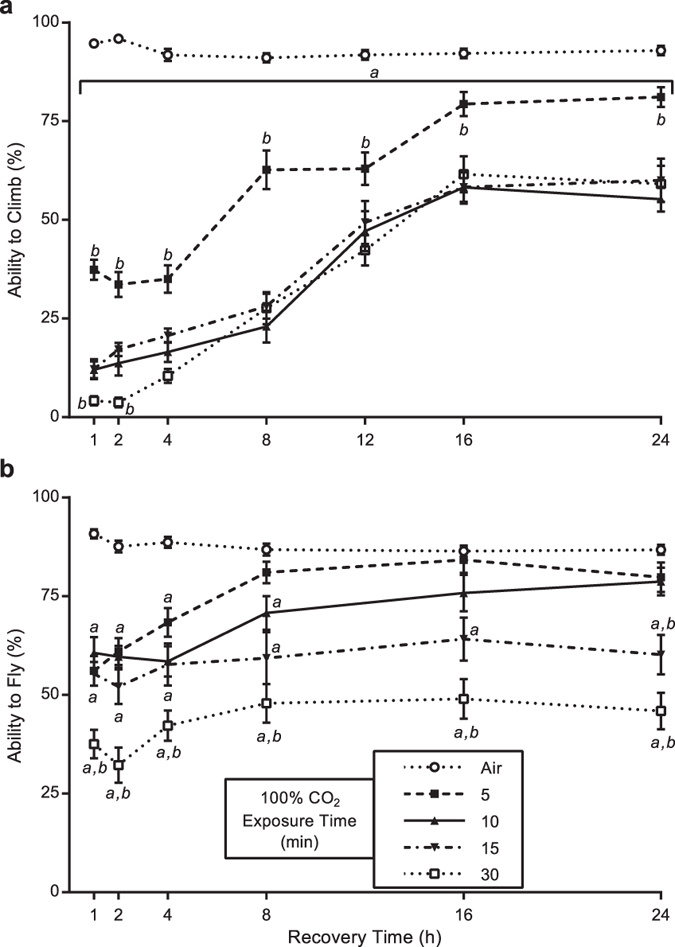
*Drosophila melanogaster* climbing and flight abilities are reduced by 100% CO_2_ in a dose-dependent manner. Flies were exposed to 100% CO_2_ for the times (minutes) noted in the legend and allowed to recover for varying times and assayed for the ability to climb and fly. (**a**) CO_2_ exposure inhibited climbing at all recovery time points assayed. Compared to the flies that were exposed for 10 minutes, the five minute exposure flies climbed better at all recovery time points, while the 30 minute exposed flies performed worse at the one and two hour recovery time points. (**b**) Flight was reduced in flies exposed to 100% CO_2_ for five minutes for up to four hours, and for 8 hours in flies exposed for 10 minutes. Flies that were exposed for 15 or 30 minutes had reduced flight at all recovery time points assayed. Compared to the flies that were exposed for 10 minutes, the 30 minute exposure flies performed worse at all recovery time points, while the 15 minute exposed flies flew less only at the 24 hour recovery time point. The Air-exposed points represent the average of all controls performed for that time point; however, the statistical analysis (Student’s *t*-test) was performed with the individual control flies for each exposure length. The data points represent the mean ± SEM of the percentage of flies able to climb or fly. *a* = P < 0.01 vs. Air control flies, *b* = P < 0.05 vs. 10 minute CO_2_ flies.

Exposure to 100% CO_2_ for 10 minutes reduced *D. melanogaster* climbing ability by 81% after being allowed to recover for one hour (Fig. 1a, P < 0.001). This reduced climbing ability persisted for at least 7 days (37% reduction, P < 0.001). The reduction in climbing exhibits a dose-dependent response, with longer CO_2_ exposure times worsening the climbing deficits (Fig. 2a, P < 0.001). Even a short, five minute exposure led to a 57% reduction in climbing after a one hour recovery (Fig. 2a, P < 0.001), which lasted for 24 hours. This experiment was performed in the Oregon-R wild-type strain. To determine if this was a strain-specific occurrence, other wild-type strains were investigated. A 10-minute exposure to 100% CO_2_ induced climbing deficits after a 1 hour recovery in the Samarkand (20.4% reduction, P < 0.05, n = 7), Swedish-C (19.9% reduction, P < 0.01, n = 16) and Lausanne (24.5% reduction, P < 0.001, n = 19) strains. This indicates that this is not a strain-specific phenomenon.

*Drosophila melanogaster* flight was negatively affected by a 10 minute exposure to 100% CO_2_; showing a 32% reduction following a one hour recovery period (Fig. 1b, P < 0.001). These effects persist for at least 8 hours. Similar to the effects on climbing behaviour, a dose-dependent effect of CO_2_ exposure is observed on the recovery of flight ability (Fig. 2b). While the negative effect of a five minute CO_2_ exposure is absent by 8 hours, flies that were exposed for 15 minutes do not recover by 24 hours (Fig. 2b, P < 0.01). The flight deficit caused by a 15 minute CO_2_ exposure at 24 hours was reduced by an additional 14% when the exposure time was increased to 30 minutes (Fig. 2b, P < 0.05).

### Anoxia reduces *D. melanogaster* flight but not climbing

**Figure 3:**
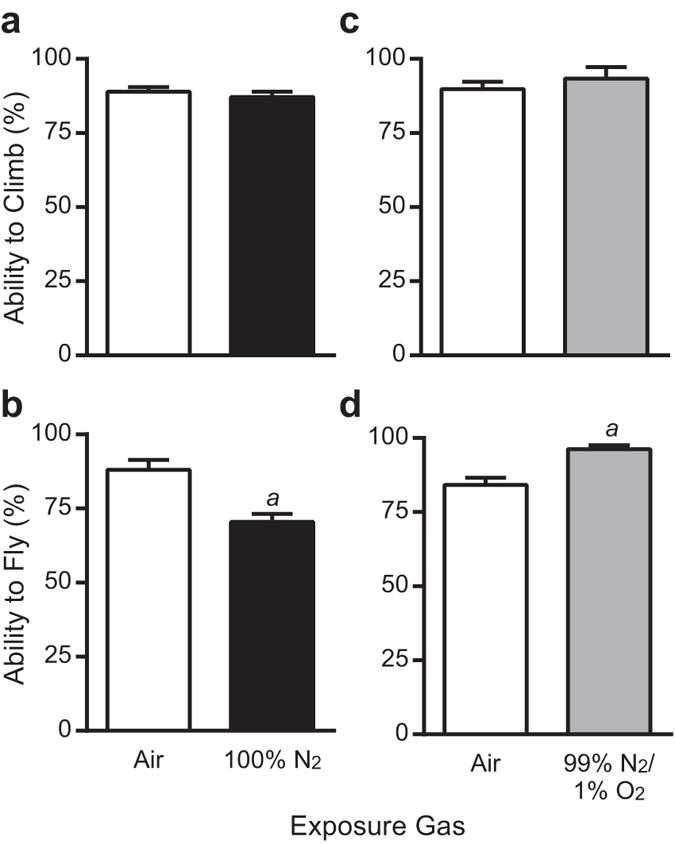
A 10 minute exposure to 100% N_2_ reduces *D. melanogaster* flight but not climbing, which is rescued by 1% O_2_. Flies were exposed to 100% N_2_ or 99% N_2_/1% O_2_ for 10 minutes and recovered for one hour prior to being assayed for climbing or flight. (**a**) Climbing ability is unaffected by the N_2_ exposure; however, (**b**) flight is reduced. (**c**) Climbing remains unaffected by 99% N_2_ exposure, while (**d**) the flight deficit is rescued by the addition of 1% O_2_. Columns represent the mean ± SEM of the percentage of flies able to climb or fly. Statistical analysis was performed by Student’s *t*-tests. *a* = P < 0.001.

To test whether the effect of CO_2_ was due to anoxia, flies were exposed to 100% N_2_ and their climbing and flight were assayed following a one hour recovery period. Following a 10 minute exposure to 100% N_2_, flight ability was reduced by 18% (Fig. 3b, P < 0.001), while climbing was unaffected (Fig. 3a). However, there was no detrimental effect on flight or climbing with a 10 minute exposure to a 99% N_2_/1% O_2_ mix (Fig. 3c,d).

### Both severe and moderate CO_2_ levels reduce *D. melanogaster* climbing

**Figure 4:**
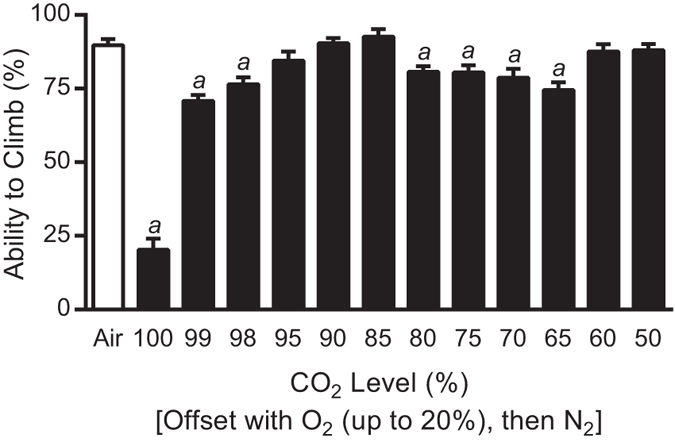
Two different ranges of CO_2_ levels adversely affect *D. melanogaster* climbing ability. Flies were exposed to varying levels of CO_2_ for 10 minutes prior to a one hour recovery. The CO_2_ mixes were initially offset by O_2_ up to 20% and then with N_2_. Bars represent the mean ± SEM of the percentage of flies able to climb. The Air-exposed control bar represents the average of all controls performed for this experiment, while the statistical analysis (Student’s *t*-test) was performed between the specific CO_2_ level and their individual air-exposed flies. *a* = P < 0.01.

Different levels of CO_2_ balanced with O_2_ and N_2_ were administered to *D. melanogaster*. Again, exposure to 100% CO_2_ led to a marked reduction in climbing ability of *D. melanogaster* (Fig. 4). A reduction in climbing ability when compared to their air-exposed controls was observed at both 99% CO_2_/1% O_2_ and 98% CO_2_/2% O_2_ mixes (P < 0.001), but was absent at 95% CO_2_/5% O_2_. Further reduction in CO_2_ levels to 85% had no detrimental effect on climbing; however, when CO_2_ levels were reduced to 80–65% [80% CO_2_/20% O_2_, 75% CO_2_/20% O_2_/5% N_2_, 70% CO_2_/20% O_2_/10% N_2_, 65% CO_2_/20% O_2_/15% N_2_], climbing was reduced again (P < 0.01). Below the 65% CO_2_ level, gas treatment no longer had any effect on climbing. Fly behaviour varied significantly over the range of CO_2_ mixes used. At the 50% CO_2_ level, the gas does not anaesthetize the flies; however, their movement slows considerably for as long as they are being exposed (Supplementary Movie 1). At higher levels of CO_2_ (100-85%), the flies are anaesthetized completely, with anaesthesia occurring faster at higher concentrations (Supplementary Movie 2). However, the middle range of CO_2_ levels (85–65%) causes flies to lose their ability to maintain their elevated position on the vial as they climb. This makes them drop down and climb repeatedly, or drop down and exhibit uncoordinated behaviour with erratic leg and wing motions (Supplementary Movie 3).

### CO_2_ levels on anaesthetizing pads can affect *D. melanogaster* climbing

**Figure 5:**
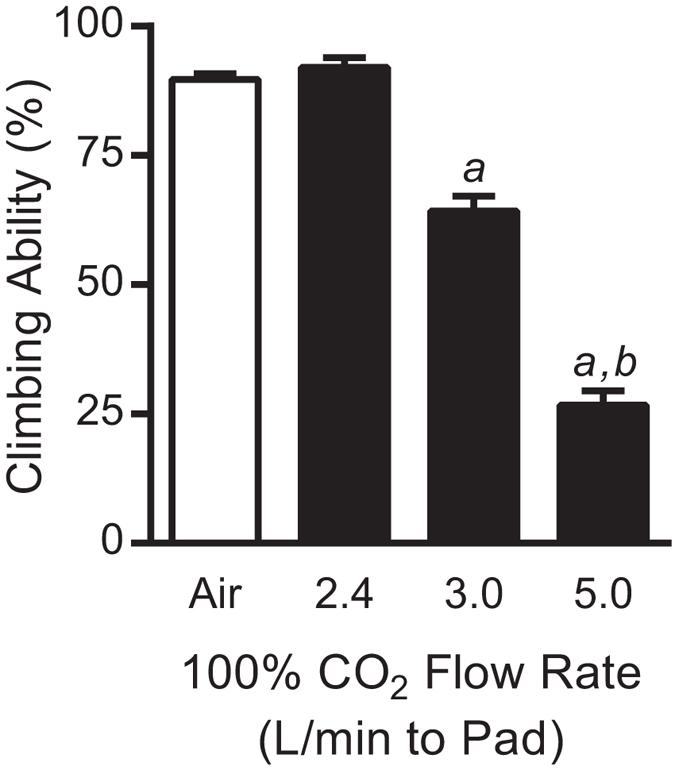
CO_2_ flow rate through a CO_2_ pad reduces *D. melanogaster* climbing ability in a flow rate-dependent manner. Flies were exposed to varying levels of CO_2_ on a CO_2_ pad with varying flow rates and allowed to recover for one hour prior to being assayed for climbing. 2.4 L/minute is the minimum CO_2_ flow rate found to anaesthetize flies and has no effect on subsequent climbing ability. However, increasing the flow to 3.0 and 5.0 L/minute decreased the ability to climb in a flow rate-dependent manner. Bars represent the mean ± SEM of the percentage of flies able to climb. The Air-exposed control bar represents the average of all controls performed for this experiment. Statistical analysis was performed by Student’s *t*-tests between the individual Air control flies and each CO_2_ flow rate. *a* = P < 0.001 vs. the Air controls and *b* = P < 0.001 vs. the 3.0 L/minute CO_2_ flies.

To better mimic laboratory conditions, a CO_2_ pad was used to expose flies to different flow rates, in contrast to the precisely controlled exposure apparatus used above. Using this method, the minimal flow necessary to anaesthetize flies was determined empirically to be 2.4 L/minute. When flies were exposed to CO_2_ for 10 minutes at a flow rate of 2.4 L/minute, and allowed to recover for one hour, there was no detrimental effect on climbing ability (Fig. 5). However, a minor increase in flow rate to 3.0 L/minute caused a 27% reduction in climbing ability (P < 0.001). This worsened to a 62% reduction in climbing when the CO_2_ flow rate was increased to 5.0 L/minute (P < 0.001).

### Flies recover from a 10 minute exposure to 99% CO_2_/1% O_2_ significantly faster than to 100% CO_2_

**Figure 6:**
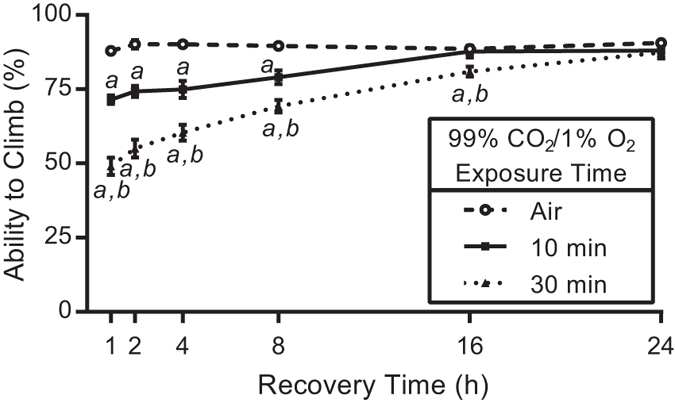
Exposure to 99% CO_2_ reduces *D. melanogaster* climbing ability for an extended period of time in a dose-dependent manner. Flies that were exposed to 99% CO_2_/1% O_2_ in the exposure apparatus had reduced climbing abilities at various recovery time points. Flies exposed for 10 minutes had reduced climbing through 8 hours, while the climbing of flies that were exposed for 30 minutes was reduced through 16 hours. The 30 minute exposure inhibited *D. melanogaster* climbing more than the 10 minute exposure. The Air-exposed points represent the average of all controls performed for that time point; however, the statistical analysis (Student’s *t*-test) was performed with the individual control flies for each exposure length. The data points represent the mean ± SEM of the percentage of flies able to climb. *a* = P < 0.01 vs. Air control flies, *b* = P < 0.01 vs. 10 minute CO_2_ flies.

When flies were exposed to a 99% CO_2_/1% O_2_ mix in the exposure apparatus and allowed to recover for one hour, their climbing ability was reduced by 17% (Fig. 6, P < 0.001). Unlike 100% CO_2_, the negative effect of 99% CO_2_/1% O_2_ on climbing is absent by 16 hours. By increasing the 99% CO_2_/1% O_2_ exposure time to 30 minutes, there is a greater reduction in climbing (39% at one hour, P < 0.001) that takes 24 hours to recover from.

## Discussion

This study represents the first comprehensive analysis of the effects of CO_2_ exposure and anoxia on *D. melanogaster* climbing and flight behaviours using precise gas mixtures. The results of the study reveal: 1) there is a chronic negative effect of a short exposure to 100% CO_2_ on climbing (Fig 1a), which is potentially indefinite in length; 2) there is a long-lasting effect of 100% CO_2_ exposure on flight for CO_2_ exposures of 15 minutes or longer (Fig. 2); and 3) the mechanism behind these detrimental effects is a combination of a CO_2_-specific mechanism and an anoxic effect (Figs 1 and 3). Although there a number of studies that have shown negative effects of CO_2_ on *D. melanogaster*, most notably on mating and reproduction^2^,^7^, negative long-term effects of CO_2_ and anoxia on *D. melanogaster* climbing and flight have not been noted or observed before, to the best of our knowledge^6^,^16^. Indeed, it is thought that there are no lasting effects from up to four hours of anoxia on *D. melanogaster*^17^,^18^,^19^, although some researchers have specifically avoided the use of CO_2_ anaesthesia prior to performing assays for these behaviours^20^. Unfortunately, the majority of methods described for analysing climbing or flight either mention the use of CO_2_ anaesthesia prior to performing the assays or make no mention of it at all^21^,^22^,^23^,^24^,^25^,^26^,^27^,^28^,^29^. When potential adverse effects of CO_2_ anaesthesia are discussed in reference to these and other behaviours, a 24 hour waiting period has been suggested to allow for sufficient recovery^30^,^31^. The results from this study definitively show that flies do not recover normal climbing ability in 24 hours if they experience an exposure to 100% CO_2_ for as little as five minutes, nor do they recover normal flight in 24 hours if exposed for 15 minutes (Fig. 2). In fact, a single 10 minute exposure of 100% CO_2_ can affect climbing ability for up to 7 days post-exposure (Fig. 1). These findings have wide ranging impacts for the *D. melanogaster* research community when analysing not just climbing and flight behaviours, but any assay that requires coordinated movement, such as mating. In a broader scope, this study provides sufficient detailed analysis of significant lasting effects in *D. melanogaster* to warrant concern for using either CO_2_ or anoxia prior to performing behavioural assays in insects. Therefore, the length of exposure to CO_2_ should be minimized and avoided if possible. Additionally, for any study involving insects, it is essential to understand the impacts of CO_2_ anaesthesia on the specific insect system being analysed before carrying out behavioural and physiological assays.

The mechanism behind the anaesthetic effect of CO_2_ and its general physiological impacts is poorly understood. However, it has been shown that CO_2_ directly inhibits glutamate receptors^12^,^32^, which function like the mammalian nicotinic receptors on motor end plates. This would be a direct mechanism to inhibit muscle activity in *D. melanogaster.* A less likely possibility could be the activation of the antennal CO_2_ receptor, which is composed of the Gr21a and Gr63a chemosensory receptors^33^. However, these receptors are likely saturated at CO_2_ levels much lower than those required for anaesthesia^34^. Neither of these mechanisms sufficiently explains the disparity between the effect of CO_2_ on climbing and flight behaviour. However, the fact that the deficits last up to one week after exposure argues that long-term changes have occurred or damage has been done that is potentially irreversible.

While it appears that the negative effects on climbing caused by anoxia and CO_2_ are through separate mechanisms, it is clear that anoxia is responsible for the long-lasting consequences observed with 100% CO_2_ exposure, since flies exposed to 99% CO_2_/1% O_2_ recover by 16 hours (Fig. 6). Climbing is unaffected by anoxia following a one hour recovery period (Fig. 3); however, this time point displays the strongest deficit with 100% CO_2_ exposure (Fig. 1). This indicates that acute behavioural effects are likely due to a CO_2_-specific mechanism, while the anoxic effect takes longer to manifest. It appears that the anoxic effect cannot be produced by severe hypoxia, as 1% O_2_ abolishes the detrimental effect on flight (Fig. 3). This is interesting because this level of hypoxia is extreme. While flies can survive short exposures to anoxia, flies cannot survive in 4% O_2_ without conditioning^35^,^36^. Anoxia leads to the spiracles remaining open, which would increase tissue exposure to high CO_2_^37^,^38^,^39^. However, low (O_2_) concentrations allow flies to continue to ventilate and close their spiracles, which would then mitigate exposure to CO_2_ at the tissue level, thus allowing them to recover faster (Fig. 6). Anoxia contributes to tissue damage by increasing the mitochondrial production of reactive oxygen species^40^,^41^,^42^ and there is strong evidence that low (O_2_) leads to increased oxidative damage in insects^43^,^44^. These mechanisms could be driving the long-term effects and lack of recovery.

The use of the exposure apparatus to treat multiple groups of flies evenly and precisely to gases is difficult to translate to a typical *D. melanogaster* laboratory. Flies are frequently anaesthetized by CO_2_ pads that are composed of flat, porous surfaces that allow for diffusion of CO_2_. It is difficult to ascertain what CO_2_ level flies are actually being exposed to in the boundary layer between the diffusing CO_2_ and the environmental air on the CO_2_ pad. However, it has been previously shown that the use of a CO_2_ pad can alter response to anaesthetics in flies^8^. In order to relate the results from the precise exposure apparatus to actual practice in research laboratories, a CO_2_ pad was incorporated into the study. 100% CO_2_ delivered at a flow rate of 2.4 L/minute was the minimum level needed to anaesthetize flies on the pad. Typically, when the CO_2_ flow rate through a CO_2_ pad is insufficient to anaesthetize flies (i.e., <2.4 L/minute), most users increase the flow enough to attain a more thorough and rapid anaesthetization. This likely leads to flow rates similar to 5.0 L/minute, or higher, which is closer to the 100% CO_2_ data from the precisely controlled experiments (compare Figs 4 and 5). However, some users might only turn up the flow rate slightly (e.g., 3.0 L/minute), which is more approximate to the precise 99% CO_2_ level. Given these similarities, minimal changes in flow rates will yield dramatically different impacts on fly behaviour. In most laboratories CO_2_ flow rates are set by “ear,” that is, the flow is turned up until the CO_2_ is heard to be flowing through the CO_2_ pad. It is difficult to ascertain what flow rate this equates to. An 11 L/minute flow rate (the maximum the mass flow controllers allowed) was undetectable to our hearing, which is likely to be very close to a 100% CO_2_ exposure. In respect to practices in *D. melanogaster* research laboratories, we suggest that modification of behavioural assays to account for this CO_2_ effect is completely possible without any additional equipment for monitoring flow or mixing gases. Since the minimal flow necessary to anaesthetize *D. melanogaster* (2.4 L/minute) had no obvious negative effects on climbing, it is possible that if caution is exhibited, negative consequences can be avoided.

This study clearly demonstrates a vital need for *D. melanogaster* researchers to use CO_2_ anaesthesia with greater caution. This has already been the standard of practice for research on mating behaviour, which has been shown to be affected by CO_2_^2^,^45^. Our results encourage *D. melanogaster* researchers to take the same careful approach with any *D. melanogaster* behavioural assay. This care needs to be exercised in each individual laboratory, as the setups are unique and the CO_2_ delivery to the fly might be very different. We suggest that each laboratory carefully take into account the amount of CO_2_ that can be delivered to the fly without negative consequences on their behavioural assay. Or, conversely, determine the minimum time needed for the flies to recover from the effects of CO_2_ exposure. Additionally, since there were marked differences in the negative effects of CO_2_ exposure in flies exposed for five minutes versus 10 minutes (Fig. 2), it is also very important that control flies be exposed to the same amount of CO_2_ as experimental flies. To work around this potential impact of CO_2_, many members of the fly community already use cold immobilisation as a method to avoid CO_2_ exposure. However, this method has also been shown to affect some behaviours and physiology of *D. melanogaster*^2^,^46^,^47^. Therefore, we caution against the untested use of any form of anaesthesia or immobilisation in general.

This study shows that 100% CO_2_ exposure has profound and long-lasting impacts on *D. melanogaster* climbing and flight. Furthermore, the mechanism behind this effect is a CO_2_-specific mechanism separate from an additional anoxic effect. In so doing, it conclusively demonstrates that both CO_2_ and anoxia can markedly affect behavioural, and likely physiological, studies in *D. melanogaster*. We therefore advocate a more careful and considered use of any anaesthetic or immobilisation technique in respect to insect behavioural and physiological research. By monitoring the duration of exposure time, flow rate, or concentration of CO_2_ the insects are exposed to, many of these impacts may be able to be minimized. Not only is this an issue for *D. melanogaster,* but it has the potential to be important to a wide variety of insect systems. Therefore, it would be worthwhile to test the effects of CO_2_ anaesthesia more broadly across a wide range of species.

## Methods

### Fly stocks and maintenance

Wild-type strains, Oregon-R, Samarkand, Lausanne and Swedish-C were obtained from the Bloomington *Drosophila* Stock Center, Indiana University, Bloomington, IN. The Oregon-R strain was used for all experiments shown in the figures. Flies were maintained at 25 °C on Nutri-Fly^TM^ MF media (Genesee Scientific®, San Diego, CA). All tests were performed with flies no older than 20 days post eclosion.

### Exposure to gas mixtures

Flies were exposed to a variety of gas mixtures using a custom-built exposure apparatus (Supplementary Fig. 1), which distributes various gas mixtures rapidly and evenly to multiple vials of flies at set flow rates. The flies were kept in a polypropylene fly vial (Genesee Scientific) that had the bottom replaced with a stainless steel wire mesh (W. W. Grainger® Inc., Lake Forest, IL; wire diameter of 0.012 inches and a mesh opening of 0.0213 inches) that was fused to the vial by heat. Flies were collected in groups of approximately 50 by weight (0.03–0.06 g). Four of these vials containing flies were connected in a series via silicone tubing (3/4 inch inner diameter, 1/8 inch wall thickness; United States Plastic Corp., Lima, OH). The series of vials were connected to a gas source regulated by mass flow controllers (Brooks® Instruments models 5850i and 5841A, Tylan General® model FC-2900v and Sable Systems International® version 1.1 mass flow control electronics unit). For all experiments where flow was constant, flow was maintained at 3.0 L/minute. 100% CO_2_ administered at this flow rate was found to cause rapid anaesthesia of the flies in the vials (Supplementary Movie 2). The time delay for anaesthesia to occur in the first vial to the last vial was <2 seconds. The test gas mixture was administered to the flies for the length of time indicated and immediately flushed out of the exposure apparatus with compressed air at 3.0 L/minute for 10 minutes. The flies were then transferred to fresh food vials and maintained at 25 °C for the remainder of the recovery time. Control flies were treated in the same manner, except they were exposed to compressed air instead of the experimental gas mixes. All gas exposures were performed with an inline humidifier to prevent desiccation (Supplementary Fig. 1).

Another set of experiments was carried out on a fly CO_2_ anaesthetizing pad (FlyStuff Flypad with a flowbed frame, Genesee Scientific). The CO_2_ pad replaced the vials in the exposure apparatus setup, so that flow, humidification and other variables could be controlled. The flies were counted on the pad and sorted into separate vials following the air flush. The compressed air flush was performed until the flies were visibly moving (typically <10 minutes) on the pad and quickly transferred into their recovery food vial. Control flies were held against the CO_2_ pad by an empty vial, since compressed air does not anaesthetize the flies.

### Climbing assay

Flies were subjected to the experimental gases and allowed to recover for the various times and then subjected to a climbing assay previously described by Chambers *et al.*^48^. Groups of ten flies were placed in an empty climbing vial and then tapped down to the bottom. They were allowed 18 seconds to climb past a dotted line marked 5 cm from the bottom of the vial. The number of flies above the 5 cm mark at 18 seconds was recorded as a percentage of flies able to climb/vial. In all assays, flies were transferred to a new food vial the day before the climbing assay was performed to help reduce wet food from inhibiting their climbing ability. A minimum of 8 separate trials were run per condition.

### Flight assay

The flight assay was performed as described in Chambers *et al.*^48^. Individual flies from a vial of approximately 10 flies were dropped into a clear acrylic flight box (28 cm × 28 cm × 28 cm) through a 3 cm entry hole in the center of the top. A fly was determined to be capable of flight if it maintained a steady elevation and flew in a controlled manner after being dropped. Data were collected as the percentage of flies able to fly/vial with at least 10 separate vials run per condition.

### Statistical analysis

All statistical analysis was performed on the arcsin of the square root of the ratio of flies able to climb or fly. For the majority of analyses, Student’s *t*-tests were performed between the compressed air control flies specific to the experimental group. In graphs where a single control group is plotted versus different experimental conditions, the plotted control group value represents the average of all control groups run at that time point/concentration/etc. Prior to this averaging, a one-way analysis of variance was performed on the transformed data from all the combined control groups to ensure that there were no differences between the control group values. However, the presented statistical analysis is always comparing the experimental group to its specific control. All statistics were run using *Graphpad Software Prism® Version 8* or *Microsoft® Excel 2010*.

## Additional Information

**How to cite this article**: Bartholomew, N. R. *et al.* Impaired climbing and flight behaviour in *Drosophila melanogaster* following carbon dioxide anaesthesia. *Sci. Rep.* **5**, 15298; doi: 10.1038/srep15298 (2015).

## Supplementary Material

Supplementary Information

Supplementary Movie 1

Supplementary Movie 2

Supplementary Movie 3

## References

[b1] Nilson T. L., Sinclair B. J. & Roberts S. P. The effects of carbon dioxide anesthesia and anoxia on rapid cold-hardening and chill coma recovery in Drosophila melanogaster. J. Insect Physiol. 52, 1027–1033, doi: 10.1016/j.jinsphys.2006.07.001 (2006). 16996534 PMC2048540

[b2] Barron A. B. Anaesthetising Drosophila for behavioural studies. J. Insect Physiol. 46, 439–442, doi: 10.1016/S0022-1910(99)00129-8 (2000). 12770207

[b3] Nicolas G. & Sillans D. Immediate and latent effects of carbon dioxide on insects. Annu. Rev. Entomol. 34, 97–116, doi: 10.1146/annurev.en.34.010189.000525 (1989).

[b4] Turner S. L. & Ray A. Modification of CO_2_ avoidance behaviour in Drosophila by inhibitory odorants. Nature 461, 277–281, doi: 10.1038/nature08295 (2009). 19710651

[b5] Milton C. C. & Partridge L. Brief carbon dioxide exposure blocks heat hardening but not cold acclimation in Drosophila melanogaster. J. Insect Physiol. 54, 32–40, doi: 10.1016/j.jinsphys.2007.08.001 (2008). 17884085

[b6] van Dijken F. R., van Sambeek M. J. P. W. & Scharloo W. Influence of anaesthesia by carbon dioxide and ether on locomotor activity in Drosophila melanogaster. Experientia 33, 1360–1361, doi: 10.1007/BF01920181 (1977).

[b7] Perron J. M., Huot L., Corrivault G. W. & Chawla S. S. Effects of carbon dioxide anaesthesia on Drosophila melanogaster. J. Insect Physiol. 18, 1869–1874, doi: 10.1016/0022-1910(72)90157-6 (1972). 4627650

[b8] Dawson A. G., Heidari P., Gadagkar S. R., Murray M. J. & Call G. B. An airtight approach to the inebriometer: from construction to application with volatile anesthetics. Fly (Austin) 7, 112–117, doi: 10.4161/fly.24142 (2013). 23579237 PMC3732330

[b9] Woodring J. P., Clifford C. W., Roe R. M. & Beckman B. R. Effects of CO_2_ and anoxia on feeding, growth, metabolism, water-balance, and blood composition in larval female house crickets, Acheta domesticus. J. Insect Physiol. 24, 499–509, doi: 10.1016/0022-1910(78)90051-3 (1978).

[b10] Branscome D. D., Koehler P. G. & Oi F. M. Influence of carbon dioxide gas on German cockroach (Dictyoptera: Blattellidae) knockdown, recovery, movement and feeding. Physiol. Entomol. 30, 144–150, doi: 10.1111/j.1365-3032.2005.00439.x (2005).

[b11] Tanaka A. Further studies on the multiple effects of carbon dioxide anesthesia in the German cockroach, Blattella germanica. Growth 49, 293–305 (1985). 3936753

[b12] Badre N. H., Martin M. E. & Cooper R. L. The physiological and behavioral effects of carbon dioxide on Drosophila melanogaster larvae. Comp. Biochem. Physiol. A Mol. Integr. Physiol. 140, 363–376, doi: 10.1016/j.cbpb.2005.01.019 (2005). 15792602

[b13] Bierbower S. M. & Cooper R. L. The mechanistic action of carbon dioxide on a neural circuit and NMJ communication. J. Exp. Zool. A Ecol. Genet. Physiol. 319, 340–354, doi: 10.1002/jez.1798 (2013). 23630163

[b14] Zhou D. & Haddad G. G. Genetic analysis of hypoxia tolerance and susceptibility in Drosophila and humans. Annu. Rev. Genomics Hum. Genet. 14, 25–43, doi: 10.1146/annurev-genom-091212-153439 (2013). 23808366 PMC12990993

[b15] Azad P., Zhou D., Zarndt R. & Haddad G. G. Identification of genes underlying hypoxia tolerance in Drosophila by a P-element screen. G3 (Bethesda) 2, 1169–1178, doi: 10.1534/g3.112.003681 (2012). 23050227 PMC3464109

[b16] Krishnan S. N., Sun Y.-A., Mohsenin A., Wyman R. J. & Haddad G. G. Behavioral and electrophysiologic responses of Drosophila melanogaster to prolonged periods of anoxia. J. Insect Physiol. 43, 203–210, doi: 10.1016/S0022-1910(96)00084-4 (1997). 12769903

[b17] Milton S. L. & Dawson-Scully K. Alleviating brain stress: what alternative animal models have revealed about therapeutic targets for hypoxia and anoxia. Future Neurol. 8, 287–301, doi: 10.2217/fnl.13.12 (2013). 25264428 PMC4174394

[b18] Farahani R. & Haddad G. G. Understanding the molecular responses to hypoxia using Drosophila as a genetic model. Respir. Physiol. Neurobiol. 135, 221–229, doi: 10.1016/S1569-9048(03)00049-1 (2003). 12809621

[b19] Van Voorhies W. A. Metabolic function in Drosophila melanogaster in response to hypoxia and pure oxygen. J. Exp. Biol. 212, 3132–3141, doi: 10.1242/jeb.031179 (2009). 19749106 PMC2742449

[b20] Sokol N. S., Xu P. Z., Jan Y. N. & Ambros V. Drosophila let-7 microRNA is required for remodeling of the neuromusculature during metamorphosis. Genes Dev. 22, 1591–1596, doi: 10.1101/Gad.1671708 (2008). 18559475 PMC2428057

[b21] Gargano J. W., Martin I., Bhandari P. & Grotewiel M. S. Rapid iterative negative geotaxis (RING): a new method for assessing age-related locomotor decline in Drosophila. Exp. Gerontol. 40, 386–395, doi: 10.1016/j.exger.2005.02.005 (2005). 15919590

[b22] Toma D. P., White K. P., Hirsch J. & Greenspan R. J. Identification of genes involved in Drosophila melanogaster geotaxis, a complex behavioral trait. Nat. Genet. 31, 349–353, doi: 10.1038/ng893 (2002). 12042820

[b23] Ganetzky B. & Flanagan J. R. On the relationship between senescence and age-related changes in two wild-type strains of Drosophila melanogaster. Exp. Gerontol. 13, 189–196, doi: 10.1016/0531-5565(78)90012-8 (1978). 99324

[b24] Karres J. S., Hilgers V., Carrera I., Treisman J. & Cohen S. M. The conserved microRNA miR-8 tunes atrophin levels to prevent neurodegeneration in Drosophila. Cell 131, 136–145, doi: 10.1016/j.cell.2007.09.020 (2007). 17923093

[b25] Banerjee S., Lee J., Venkatesh K., Wu C. F. & Hasan G. Loss of flight and associated neuronal rhythmicity in inositol 1,4,5-trisphosphate receptor mutants of Drosophila. J. Neurosci. 24, 7869–7878, doi: 10.1523/JNEUROSCI.0656-04.2004 (2004). 15356199 PMC1289272

[b26] Vigoreaux J. O., Hernandez C., Moore J., Ayer G. & Maughan D. A genetic deficiency that spans the flightin gene of Drosophila melanogaster affects the ultrastructure and function of the flight muscles. J. Exp. Biol. 201, 2033–2044 (1998). 9622575 10.1242/jeb.201.13.2033

[b27] Ali Y. O., Escala W., Ruan K. & Zhai R. G. Assaying locomotor, learning, and memory deficits in Drosophila models of neurodegeneration. J. Vis. Exp., (49), e2504, doi: 10.3791/2504 (2011). PMC3197301 21445036

[b28] Slawson J. B., Kim E. Z. & Griffith L. C. High-resolution video tracking of locomotion in adult Drosophila melanogaster. J. Vis. Exp., (24), e1096, doi: 10.3791/1096 (2009). PMC2762895 19390509

[b29] Williamson R. L. Lithium stops hereditary shuddering in Drosophila melanogaster. Psychopharmacology 76, 265–268, doi: 10.1007/BF00432558 (1982). 6808547

[b30] Babcock D. T. & Ganetzky B. An improved method for accurate and rapid measurement of flight performance in Drosophila. J. Vis. Exp., (84), e51223, doi: 10.3791/51223 (2014). 24561810 PMC4089396

[b31] Greenspan R. J. Fly Pushing: The Theory And Practice Of Drosophila Genetics. 2nd ed, (Cold Spring Harbor Laboratory Press, 2004).

[b32] Brosnan R. J. & Pham T. L. Carbon dioxide negatively modulates N-methyl-D-aspartate receptors. Br. J. Anaesth. 101, 673–679, doi: 10.1093/bja/aen266 (2008). 18791188

[b33] Jones W. D., Cayirlioglu P., Kadow I. G. & Vosshall L. B. Two chemosensory receptors together mediate carbon dioxide detection in Drosophila. Nature 445, 86–90, doi: 10.1038/nature05466 (2007). 17167414

[b34] Kwon J. Y., Dahanukar A., Weiss L. A. & Carlson J. R. The molecular basis of CO_2_ reception in Drosophila. Proc. Natl. Acad. Sci. USA 104, 3574–3578, doi: 10.1073/pnas.0700079104 (2007). 17360684 PMC1805529

[b35] Zhou D. *et al.* Experimental selection for Drosophila survival in extremely low O_2_ environment. PLoS One 2, e490, doi: 10.1371/journal.pone.0000490 (2007). 17534440 PMC1871610

[b36] Zhou D. *et al.* Mechanisms underlying hypoxia tolerance in Drosophila melanogaster: hairy as a metabolic switch. PLoS Genet. 4, e1000221, doi: 10.1371/journal.pgen.1000221 (2008). 18927626 PMC2556400

[b37] Förster T. D. & Hetz S. K. Spiracle activity in moth pupae—The role of oxygen and carbon dioxide revisited. J. Insect Physiol. 56, 492–501, doi: 10.1016/j.jinsphys.2009.06.003 (2010). 19524587

[b38] Heinrich E. C., McHenry M. J. & Bradley T. J. Coordinated ventilation and spiracle activity produce unidirectional airflow in the hissing cockroach, Gromphadorhina portentosa. J. Exp. Biol. 216, 4473–4482, doi: 10.1242/Jeb.088450 (2013). 24031063

[b39] Levy R. I. & Schneiderman H. A. Discontinuous respiration in insects—II. The direct measurement and significance of changes in tracheal gas composition during the respiratory cycle of silkworm pupae. J. Insect Physiol. 12, 83–104, doi: 10.1016/0022-1910(66)90068-0 (1966). 5902575

[b40] Vanden Hoek T. L., Becker L. B., Shao Z., Li C. & Schumacker P. T. Reactive oxygen species released from mitochondria during brief hypoxia induce preconditioning in cardiomyocytes. J. Biol. Chem. 273, 18092–18098, doi: 10.1074/jbc.273.29.18092 (1998). 9660766

[b41] Guzy R. D. *et al.* Mitochondrial complex III is required for hypoxia-induced ROS production and cellular oxygen sensing. Cell Metab. 1, 401–408, doi: 10.1016/j.cmet.2005.05.001 (2005). 16054089

[b42] Waypa G. B. & Schumacker P. T. Hypoxia-induced changes in pulmonary and systemic vascular resistance: where is the O_2_ sensor? Respir. Physiol. Neurobiol. 174, 201–211, doi: 10.1016/j.resp.2010.08.007 (2010). 20713189 PMC2991475

[b43] Zhao H. W. & Haddad G. G. Review: Hypoxic and oxidative stress resistance in Drosophila melanogaster. Placenta 32, S104–S108, doi: 10.1016/j.placenta.2010.11.017 (2011). 21353099 PMC3073591

[b44] Azad P., Ryu J. & Haddad G. G. Distinct role of Hsp70 in Drosophila hemocytes during severe hypoxia. Free Radical Biol. Med. 51, 530–538, doi: 10.1016/j.freeradbiomed.2011.05.005 (2011). 21616137 PMC3138732

[b45] Nichols C. D., Becnel J. & Pandey U. B. Methods to assay Drosophila behavior. J. Vis. Exp., (61), e3795, doi: 10.3791/3795 (2012). PMC3671839 22433384

[b46] de Crespigny F. E. C. & Wedell N. The impact of anaesthetic technique on survival and fertility in Drosophila. Physiol. Entomol. 33, 310–315, doi: 10.1111/j.1365-3032.2008.00632.x (2008).

[b47] MacAlpine J. L., Marshall K. E. & Sinclair B. J. The effects of CO_2_ and chronic cold exposure on fecundity of female Drosophila melanogaster. J. Insect Physiol. 57, 35–37, doi: 10.1016/j.jinsphys.2010.09.003 (2011). 20868691

[b48] Chambers R. P. *et al.* Nicotine increases lifespan and rescues olfactory and motor deficits in a Drosophila model of Parkinson’s disease. Behav. Brain Res. 253, 95–102, doi: 10.1016/j.bbr.2013.07.020 (2013). 23871228

