# Supplementary material for: Impaired climbing and flight behaviour in Drosophila melanogaster following carbon dioxide anaesthesia
Source: Sci Rep. 2015 Oct 19;5:15298. doi: 10.1038/srep15298 (PMC4609961; doi:10.1038/srep15298)
Supplement: Supplementary Information [file srep15298-s1.pdf]

Supplementary Information

Impaired climbing and flight behaviour in Drosophila melanogaster following carbon dioxide anaesthesia

Nathan R. Bartholomew[1], Jacob M. Burdett[1], John M. VandenBrooks[2], Michael Quinlan[2] & Gerald B. Call[3,*]

[1]Arizona College of Osteopathic Medicine (AZCOM), Midwestern University, Glendale, AZ, 85308, USA
[2]Department of Physiology, AZCOM, Midwestern University, Glendale, AZ 85308, USA
[3]Department of Pharmacology, AZCOM, Midwestern University, Glendale, AZ 85308, USA

*Correspondence should be addressed to G. B. Call (gcall@midwestern.edu)

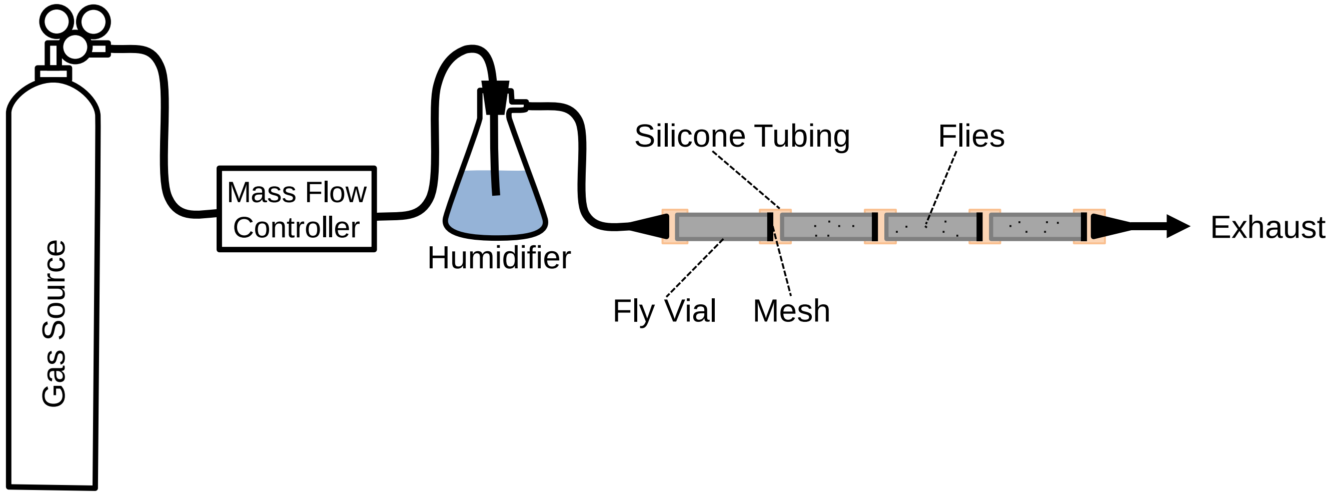

Supplementary Figure 1. Exposure apparatus design. The exposure apparatus begins with one or multiple gas sources that flow into a single or multiple mass flow controller(s) to accurately set the gas levels. Following this, the gas is humidified in a water-filled Erlenmeyer flask before continuing to the vials containing the flies. Four polypropylene vials with a wire mesh base held approximately 50 flies, which were connected in a series by silicone tubing.

Supplementary Movie 1. Behaviour of flies in response to 50% CO_2 exposure. The beginning of the movie shows flies in the exposure apparatus vial prior to 50% CO_2 exposure. The gas is turned on when the "50% CO_2 Start" text appears. It takes a few seconds for the gas to reach the flies through the exposure apparatus. When the gas reaches the flies, they slow their movement considerably, but are not anaesthetized.

Supplementary Movie 2. Behaviour of flies in response to 100% CO_2 exposure. The beginning of the movie demonstrates the normal behaviour of flies in the exposure apparatus vial prior to the 100% CO_2 exposure. The gas is turned on when the "100% CO_2 Start" text appears. All flies are immediately anaesthetized once the 100% CO_2 traverses the exposure apparatus to the vial.

Supplementary Movie 3. Behaviour of flies in response to 75% CO_2 exposure. The first part of the movie shows the flies' behaviour prior to 75% CO_2 exposure. The gas is turned on when the "75% CO_2 Start" text appears. Following the few seconds it takes for the gas to reach the flies through the exposure apparatus, their behaviour is mixed. Some flies appear to be more affected than others, but eventually all succumb to the gas. By the end of the movie (>2 minute exposure time), it appears that all flies are mostly anesthetized, but continue to show erratic leg and wing movements.
